# Comparative analysis of sodium coupled vitamin C transporter 2 in human osteoarthritis grade 1 and grade 3 tissues

**DOI:** 10.1186/1471-2474-15-9

**Published:** 2014-01-08

**Authors:** Alan R Blackburn, Mark W Hamrick, Norman Chutkan, Rajnikumar Sangani, Jennifer L Waller, Raymond Corpe, Puttur D Prasad, Carlos M Isales, Vadivel Ganapathy, Sadanand Fulzele

**Affiliations:** 1 Department of Orthopaedic Surgery, Georgia Reagents University, Augusta, GA 30912, USA; 2 Department of Cellular Biology and Anatomy, Georgia Reagents University, Augusta, GA 30912, USA; 3 Department of Biostatistics and Epidemiology, Georgia Reagents University, Augusta, GA 30912, USA; 4 Department of Biochemistry and Molecular Biology, Georgia Reagents University, Augusta, GA 30912, USA; 5 Institute of Regenerative and Reparative Medicine, Georgia Reagents University, Augusta, GA 30904, USA

**Keywords:** SVCT2, Vitamin C transporter, Articular cartilage, Osteoarthritis

## Abstract

**Background:**

Nutrient levels are known to influence the development of osteoarthritis (OA), presumably by modulating levels of matrix biosynthesis and degradation. These processes may be affected by ascorbic acid (AA), an antioxidant which acts as a cofactor for numerous biochemical reactions and is essential for post-translational modifications of collagen. In this study we examined the expression of SVCT2, the only known Sodium coupled vitamin C transporter isoform present in articular cartilage, in human articular cartilage explants derived from both normal and osteoarthritis articular cartilage.

**Methods:**

OA1 and OA3 human articular cartilage was carefully dissected and macroscopically graded for degeneration via the Collins scale. The tissue samples were histologically examined by Hematoxylin and Eosin and Safranin O and Fast Green staining. SVCT2 expression analysis was performed at mRNA level by quantitative real time PCR and at a protein level by immunohistochemistry.

**Results:**

Our quantitative real time PCR showed marked variation in the expression of SVCT2 in human osteoarthritic articular cartilage. SVCT2 expression was significantly down-regulated (p = 0.0001) in the Collins grade 3 (OA3) compared to Collins grade 1 (OA1) tissue. Furthermore, slides stained with fluorescent antibodies to SVCT2 demonstrated greatly reduced fluorescence for the SVCT2 transporter on the chondrocyte plasma membrane in the osteoarthritic tissue samples.

**Conclusions:**

These findings demonstrate that the expression of SVCT2 transporter is significantly altered in human osteoarthritic tissues (OA3). The modulation of this transporter could therefore potentially influence the prevention, management and treatment of osteoarthritis.

## Background

Osteoarthritis (OA) can be defined as a process of aberrant repair with gradual and progressive loss of articular cartilage through degradative mechanisms. Progressive deterioration of the articular cartilage leads to impaired joint motion, severe pain, and ultimately, disability. Primarily, the causes of OA are aging, obesity , mechanical stress [1], oxidative stress [2] and altered nutrient levels [3], all of which have been found to affect matrix composition, presumably by modulating rates of macromolecular biosynthesis and degradation. These processes may be modulated in part by the micronutrient ascorbic acid (vitamin C or AA), which is essential for normal collagen synthesis through its role in the post-translational modification of collagen. These post-translation processes include the hydroxylation of proline and lysine both of which are critical for the maintence of connective tissue integrity [4]. Additionally, vitamin C is the primary water-soluble antioxidant, serving as a powerful scavenger of reactive oxygen and nitrogen species. It is therefore clearly evident that an optimal supply of ascorbic acid is absolutely essential for the normal function of articular cartilage.

Since adult cartilage contains no vasculature supply, chondrocytes metabolize largely anaerobically, obtaining their nutrition via the synovial fluid by means of diffusion [5]. However, since ascorbic acid is highly water-soluble, it cannot simply diffuse across the hydrophobic lipid bilayer of the plasma membrane to gain access into these cells; specific transport systems must exist in the plasma membrane to mediate the entry process. Two isoforms of the sodium-dependent vitamin C transporters (SVCT1 and SVCT2) are expressed in many human and mouse tissues but only SVCT2 is expressed in articular cartilage [6,7]. Additionally, it has been also reported that aging, oxidative stress, and inflammatory factors regulate SVCT2 expression in various mammalian tissues, and the same factors also contribute to osteoarthritis. Therefore, it is important to collect fundamental information regarding changes in the expression of SVCT2 in osteoarthritis. We hypothesize that the SVCT2 transporter plays a key role in the normal function of articular chondrocytes, and that alteration in SVCT2 expression may contribute to cartilage degeneration. Alterations in the expression of the SVCT2 transporter have not yet been studied extensively in the osteoarthritic human tissues.

The present study examined the expression of SVCT2 in human articular chondrocytes derived from osteoarthritis grade 1 (OA1) and osteoarthritis grade 3 (OA3) at mRNA level by quantitative real time PCR and at protein level by immunohistochemistry. Our findings demonstrate that when normal human articular cartilage is compared to osteoarthritic tissue, notable changes in the distribution and expression of SVCT2 are seen. The modulation of this transporter could therefore potentially influence the prevention, management and treatment of osteoarthritis.

## Methods

### Patient samples

Osteoarthritis grade 1 (OA1) and Osteoarthritis grade 3 (OA3) human cartilage was obtained from distal, anterior, and posterior femoral cuts from the knee joints of 29 tissue donors (13 males and 16 females) undergoing total knee arthroplasty procedures. All studies were performed with approval from the Georgia Health Sciences University Institutional Review Board (IRB) and written informed consent was obtained from all participants for the publication of individual data and accompanying clinical images. The cartilage tissue was obtained from the operating room at the time of surgery, transported to the laboratory, washed with PBS, and immediately macroscopically graded for degeneration via the Collins scale. Following macroscopic grading, this tissue was assigned into one of two groups, OA1 (Collins grade 1) or OA3 (Collins grade 3–4) [8]. In each case, the groups (OA1 and OA3) were derived from a single knee of a single patient. This graded cartilage tissue was then dissected from the remnant joint tissue using a scalpel, with care taken to avoid underlying bone or tissue from osteophytes. The tissues were divided into two parts, 1st part for gene expression analysis (RNA isolation and real time PCR) and 2nd part for histological and immunohistochemical examination.

### Isolation of RNA, synthesis of cDNA, and real-time PCR

Total RNA was isolated from OA1 and OA3 grade tissues. The tissues were ground in liquid N_2_ with a mortar and pestle, dissolved in Trizol for RNA isolation, per manufacturer’s instructions, and the quality of the RNA preparations was monitored by absorbance at 260 and 280 nm (Helios-Gamma, Thermo Spectronic, Rochester, NY). The RNA was then reverse-transcribed into complementary deoxyribonucleic acid (cDNA) using iScript reagents from Bio-Rad on a programmable thermal cycler (PCR-Sprint, Thermo Electron, Milford, MA). 50 ng of cDNA was amplified in each real-time PCR reaction using a Bio-Rad iCycler, ABgene reagents (Fisher scientific) and gene specific primers for HuSVCT2-F GCAGAGCTGTTGCACACAGAA, HuSVCT2-R CGAGGAGGCCGATGACTACTT [9], HuGAPDH-F CATGAGAAGTATGACAACAGCCT HuGAPDH-R AGTCCTTCCACGATACCAAAGT accession number XM_005253678 and Hu18S-F CAGCCACCCGAGATTGAGCA, Hu18S-F TAGTAGCGACGGGCGGTGTG accession number NR_003286). Average of Glyceraldehyde-3-phosphate dehydrogenase (GAPDH) and 18S was used as the internal control for normalization.

### Histological and immunohistochemistry examination

Graded cartilage was dissected from the remnant joint tissue, separated into both healthy and osteoarthritic samples as previously described, trimmed into approximately 4 mm × 4 mm squares, embedded within OCT and snap frozen in liquid nitrogen. Tissue sections were mounted onto glass slides after sectioning with a cryotome. The three staining approaches utilized included Hematoxylin and Eosin, Safranin O and Fast Green, and immunofluorescent staining for SVCT2. For immunostaining, sections were fixed with cold acetone for 5 minutes, blocked in normal donkey serum, incubated with primary goat anti-SVCT-2 polyclonal antibodies (Santa Cruz) for 2 hrs at room temperature then washed and incubated with FITC-labeled goat anti-mouse secondary antibodies. Sections were counterstained with DAPI and mounted using aqueous medium. All staining techniques were performed on both normal and osteoarthritic cartilage and slides were qualitatively analyzed.

### Statistical analysis

All statistical analyses were performed using SAS 9.2. Statistical significance was assessed using an alpha level of 0.05 unless otherwise noted. Descriptive statistics including the mean, median and standard deviation were calculated on the mean ∆∆Ct for SVCT2 outcome measures. A one-sample t-test was used to determine whether the mean ∆∆Ct for SVCT2 was significantly different than zero.

## Results and discussion

Numerous studies have suggested that high dietary intake of ascorbic acid slows the progression of osteoarthritis, both in guinea pig [10] and human models [11]. This is intuitively understandable as ascorbic acid is required for regulation of various transcription factors and collagen synthesis. Like other higher primates, humans have to rely on dietary intake of vitamin C to satisfy the body’s daily requirements [4]. High dietary intakes of ascorbic acid alone, however, cannot account for this effect, as ascorbic acid is highly water-soluble and cannot freely diffuse into a cell.

The recent cloning and characterization of two transporters, SVCT1 and SVCT2 [12], have provided an insight into the mechanisms involved in vitamin C assimilation from the diet. Uptake of ascorbic acid into the chondrocyte is achieved primarily through the sodium dependent vitamin C transporter (SVCT2), a transporter which has been shown previously to concentrate ascorbic acid in guinea pig articular chondrocytes [6] and primary passaged human chondrocytes [7]. Because of these essential functions of SVCT2, we sought to investigate the change in the expression of SVCT2 in normal and osteoarthritic cartilage tissues. Although direct functional changes in the transporters could not be demonstrated, measurement of their expression levels could provide some insight into their relative roles during patho-physiologic changes. Initially, after dissection of healthy and osteoarthritic cartilage explants tissues, these tissues were stained and characterized using Safranin O and Fast Green or Hematoxylin and Eosin (Additional file 1: Figure S1). These techniques each supported the macroscopic grading performed prior to experimentation and clarified that we were, in fact, working with appropriately characterized cartilage tissue.

**Figure 1:**
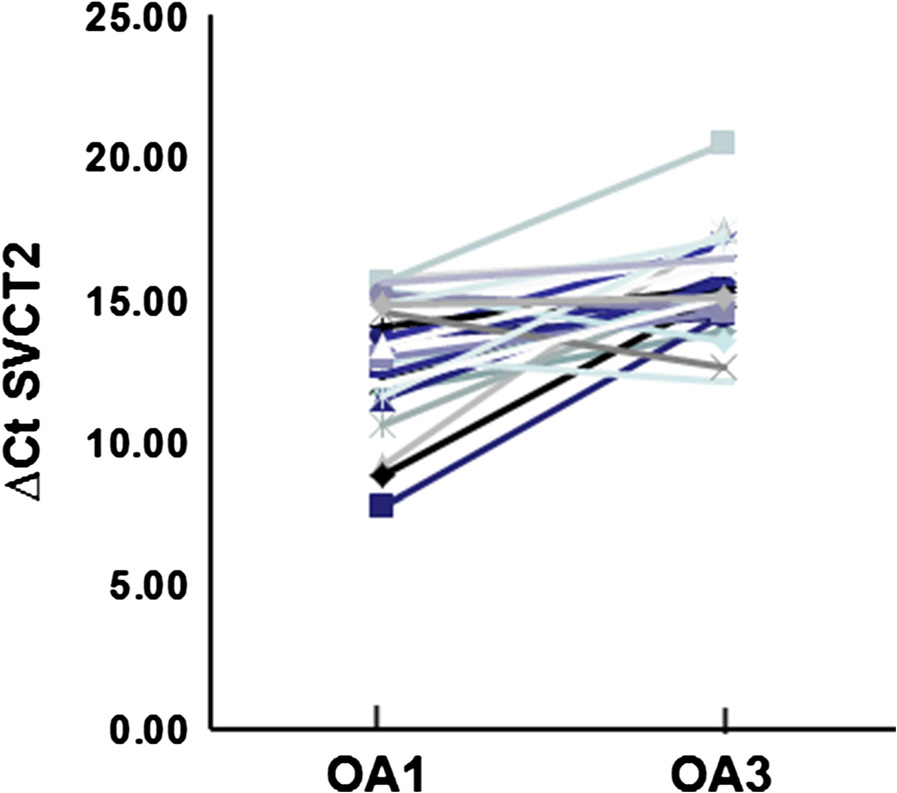
**Spaghetti plot showing decrease expression of SVCT2 in OA3 grade samples.** Y-axis showing ∆CT values [∆CT values is the difference between the CT (cycle threshold) values of the target gene (SVCT2) and a housekeeping gene] of OA1 and OA3 samples (n = 29, p < 0.0001). CT levels are inversely proportional to the amount of target in the sample (i, e the lower the CT level the greater the amount of target gene expression in the sample).

**Figure 2:**
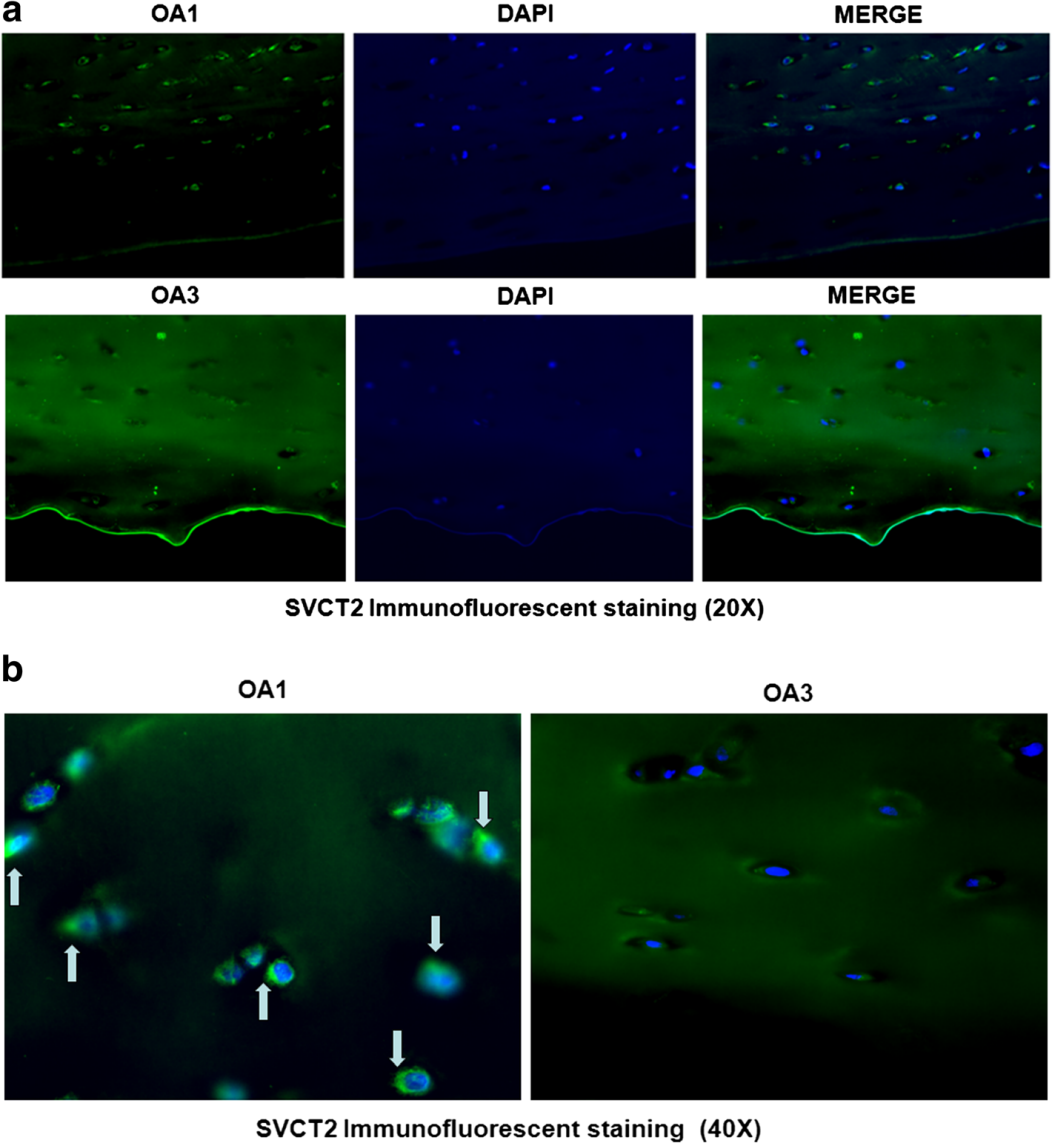
**Representative immunofluorescence localization of SVCT2 protein in OA1 and OA3 Collins scale grade human cartilage tissue. a)** 20× and **b)** 40×. The immunofluorescence analysis was performed using a polyclonal antibody specific for SVCT2. Secondary antibody was anti-goat IgG labeled with FITC. The nuclei were stained with Hoechst dye.

After examining the expression of SVCT2 mRNA from both OA1 and OA3 osteoarthritic cartilage tissues, it is clear that at an mRNA level, OA3 osteoarthritic tissue is not well equipped for optimal ascorbic acid transport (Figure 1, Additional file 2: Figure S2), as our studies revealed a statistically significant median ∆∆Ct decrease in SVCT2 expression of −2.3 (4.93-fold) when OA3 was compared to OA1. A total of 29 patient samples (Table 1) were used for this analysis, and the mean age was 66.1 years (Range 45–78, SD = 7.9). Table 2 shows the descriptive statistics for the ∆∆Ct for SVCT2 (p < 0.0001). This finding was supported by a decrease in the immunoreactivity for the SVCT2 transporter present on the plasma membrane of the osteoarthritic chondrocyte (Figure 2, Additional file 3: Figure S3). There was positive correlation between mRNA and protein level. OA3 samples showed similar pattern of down-regulation of SVCT2 at both mRNA and protein level. Down-regulation of SVCT2 expression at mRNA level leads to less SVCT2 protein synthesis. This could be the reason for less transporter activity and ultimately less in-take of vitamin C inside the cell.

**Table 1:** List of donors and ∆∆Ct values

**Sample no.**	**Sex**	**Age**	**Relative ∆∆CT Ct compared to OA1 grade**
1	F	70	−2.11
2	F	72	−6.69
3	M	62	−8.29
4	F	57	−3.01
5	F	56	−4.89
6	F	67	−0.67
7	M	69	−2.11
8	M	70	−1.76
9	F	70	−0.68
10	F	76	−6.37
11	M	70	−4.86
12	F	78	−4.26
13	F	68	−3.22
14	M	63	−3.26
15	M	74	−1.60
16	M	71	−1.19
17	M	78	0.54
18	F	61	−4.22
19	M	61	0.03
20	F	56	−0.54
21	F	55	−2.70
22	F	61	1.96
23	F	61	NA*
24	M	63	−3.35
25	M	63	0.19
26	M	77	−2.49
27	M	73	−0.88
28	F	69	−3.02
29	F	45	−1.24

NA* = No ct values/ amplification in OA3.

∆∆CT (delta delta Ct) is the difference between control and experimental samples.It is explained below the Table 2. (∆∆CT SVCT2: Different between control (OA1) ∆CT values and OA3 ∆CT values).

**Table 2:** Descriptive statistics t-test results

**Variable**	**Number of samples**	**Mean**	**SD**	**Median**	**p-value**
∆∆CT SVCT2	29	−2.5246	2.3304	−2.3000	<0.0001

CT: cycle threshold.

∆CT values: values are the difference between the CT (cycle threshold) values of the target gene and a housekeeping gene.

∆∆CT SVCT2: Different between control (OA1) ∆CT values and OA3 ∆CT values.

Though aging has been implicated as a factor contributing to decreased ascorbic acid uptake in earlier studies, [13] and all of our patients were between the ages of 45 and 78, it is important to note that aging was not the cause of our particular findings. As both the OA1 and OA3 tissues used for comparison in this study were derived from single knees of individual patients, age was not a factor in the comparisons made between grades. The findings demonstrated by the explant tissue in this study are a direct result of osteoarthritic cartilage degradation alone.

Furthermore, despite variations in the magnitude of the decreased expression, this decrease was consistently present in 86% of patients that we examined. Although the mechanisms responsible for the down-regulation of SVCT2 expression in osteoarthritic tissue were not explored in the present study, this down-regulation suggests alterations in the transcriptional regulation of SVCT2 by one or more of various factors. These factors may include oxidative stress, inflammatory factors, growth factors, growth hormones or a decline in RNA stability (e.g. by epigenetic factors such as microRNAs), all of which have been implicated in development of osteoarthritis. Recently, various reports have revealed a transcriptional control mechanism for SVCT2 activity by hormones and intracellular signalling molecules. In these studies, the SVCT2 expression was altered by epidermal growth factor and foetal bovine serum in a human trophoblast cell line [14], and by glucocorticoids [14], zinc, calcium and phosphate ions [14] in osteoblastic cells. Inflammatory factors TNF-α and IL-1ß suppressed the uptake of AA in a dose- and time-dependent manner in human endothelial cells [15]. Furthermore, this transporter is also regulated by redox modulator in human lens epithelial cells [16], C2C12 myotubes [17] and mouse bone marrow stromal cells [18]. Future studies should seek to delineate the mechanisms of interaction among the factors mentioned above with particular reference to their role in vitamin C homeostasis in the human articular chondrocyte. The outcome of such studies could help to develop improved and more effective strategies for management and treatment of osteoarthritis.

## Conclusion

The present results demonstrate that the distribution and overall expression of SVCT2 is significantly altered in degenerative human articular cartilage grade 3 (OA3) when compared to human articular cartilage grade 1 (OA1). This study therefore provides new and previously undocumented evidence regarding the regulation of SVCT2 transporter and its contribution to the osteoarthritis.

## Abbreviations

AA: Ascorbic acid; OA: Osteoarthritis; SVCT2: Sodium-dependent vitamin C transporters.

## Competing interests

The authors declare that they have no competing interests.

## Authors’ contributions

Study design: SF, MWH, and ARB II. Acquisition of data: SF, ARB II, PTP, RS, RC. Analysis and interpretation of data: SF, JLW, ARB, MWH, CMI, VG, RC. Manuscript preparation: SF, VG, MWH, ARB II , NC, CMI. All authors read and approved the final manuscript.

## Pre-publication history

The pre-publication history for this paper can be accessed here:

http://www.biomedcentral.com/1471-2474/15/9/prepub

## Supplementary Material

Additional file 1: Figure S1 (a) Representative Hematoxylin and Eosin and (b) Safranin O staining of OA1 and OA3 Collins scale grade human cartilage tissue. Click here for file

Additional file 2: Figure S2 Graph plot showing relative ∆∆CT compared to OA1 grade human cartilage tissue. Click here for file

Additional file 3: Figure S3 Quantification of SVCT2 immunofluorescence of OA1 and OA3 Collins scale grade human cartilage tissue. Values are the mean ± SEM. **P < 0.0001 by unpaired Student’s t-test. Click here for file
