# Supplementary figures and images for: Comparative analysis of sodium coupled vitamin C transporter 2 in human osteoarthritis grade 1 and grade 3 tissues
Source: BMC Musculoskelet Disord. 2014 Jan 8;15:9. doi: 10.1186/1471-2474-15-9 (PMC3922806; doi:10.1186/1471-2474-15-9)

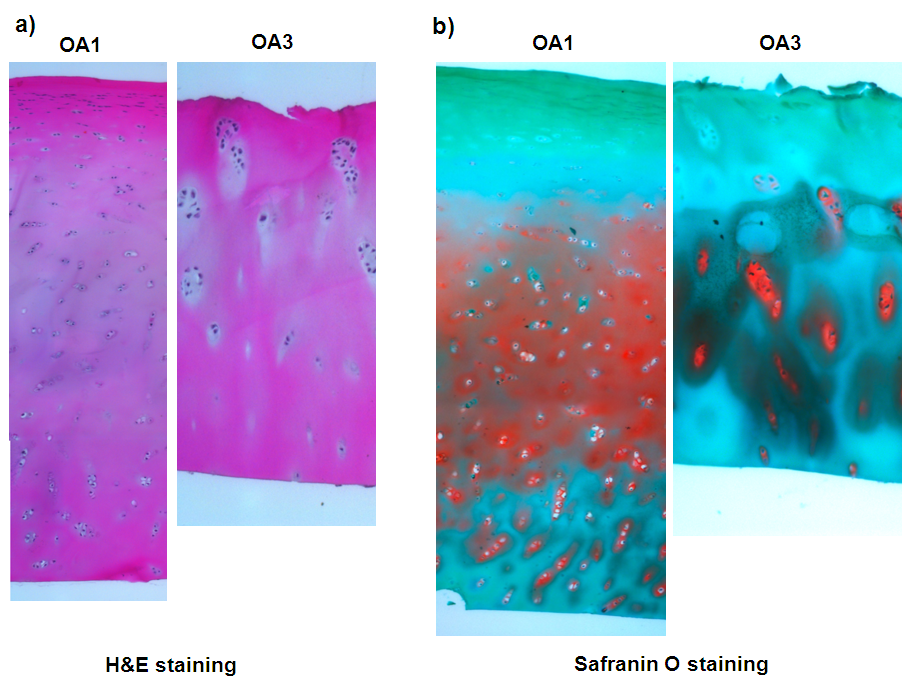

Supplement: Additional file 1: Figure S1 — (a) Representative Hematoxylin and Eosin and (b) Safranin O staining of OA1 and OA3 Collins scale grade human cartilage tissue. [file 1471-2474-15-9-S1.tiff]

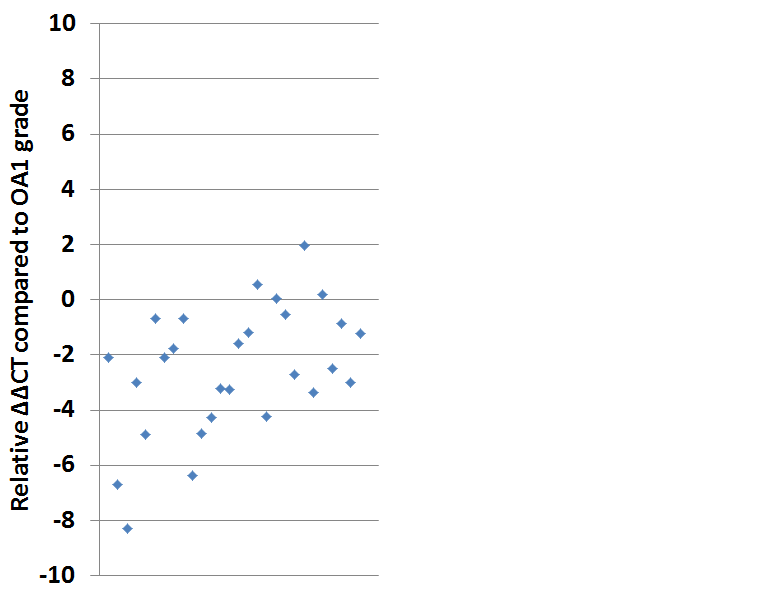

Supplement: Additional file 2: Figure S2 — Graph plot showing relative ∆∆CT compared to OA1 grade human cartilage tissue. [file 1471-2474-15-9-S2.tiff]

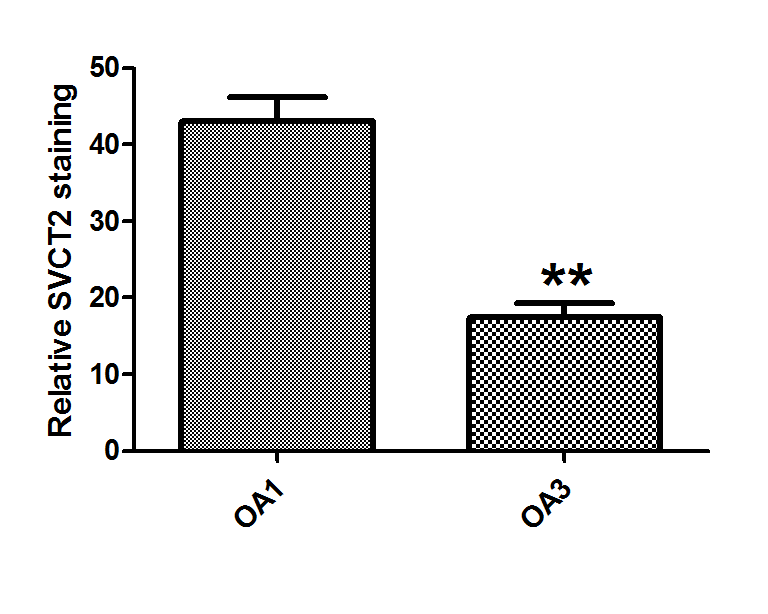

Supplement: Additional file 3: Figure S3 — Quantification of SVCT2 immunofluorescence of OA1 and OA3 Collins scale grade human cartilage tissue. Values are the mean ± SEM. **P < 0.0001 by unpaired Student’s t-test. [file 1471-2474-15-9-S3.tiff]
